# ProGeo-Neo v2.0: A One-Stop Software for Neoantigen Prediction and Filtering Based on the Proteogenomics Strategy

**DOI:** 10.3390/genes13050783

**Published:** 2022-04-28

**Authors:** Chunyu Liu, Yu Zhang, Xingxing Jian, Xiaoxiu Tan, Manman Lu, Jian Ouyang, Zhenhao Liu, Yuyu Li, Linfeng Xu, Lanming Chen, Yong Lin, Lu Xie

**Affiliations:** 1 College of Food Science and Technology, Shanghai Ocean University, Shanghai 201306, China; cyliu1997@yeah.net (C.L.); luman8@126.com (M.L.); yyli1017@163.com (Y.L.); lmchen@shou.edu.cn (L.C.); 2 Shanghai-MOST Key Laboratory of Health and Disease Genomics, Institute for Genome and Bioinformatics, Shanghai Institute for Biomedical and Pharmaceutical Technologies, 779 Old Humin Road, Shanghai 200237, China; zhang_yu_doris@163.com (Y.Z.); jianxingxing@foxmail.com (X.J.); tanxiaoxiu18@163.com (X.T.); ouyangjian12@163.com (J.O.); lzh_126e@126.com (Z.L.); linfneg@163.com (L.X.); 3 School of Health Science and Engineering, University of Shanghai for Science and Technology, Shanghai 200093, China; yong_lynn@163.com; 4 Department of Bioinformatics and Biostatistics, Shanghai Jiao Tong University, 800 Dongchuan Road, Shanghai 200240, China; 5 The Center for Bioinformatics and Computational Biology, Shanghai Key Laboratory of Regulatory Biology, The Institute of Biomedical Sciences and School of Life Sciences, East China Normal University, Shanghai 200241, China

**Keywords:** bioinformatics, neoantigen, proteogenomic, tumor immunotherapy

## Abstract

A proteogenomics-based neoantigen prediction pipeline, namely ProGeo-neo, was previously developed by our team to predict neoantigens, allowing the identification of class-I major histocompatibility complex (MHC) binding peptides based on single-nucleotide variation (SNV) mutations. To improve it, we here present an updated pipeline, i.e., ProGeo-neo v2.0, in which a one-stop software solution was proposed to identify neoantigens based on the paired tumor-normal whole genome sequencing (WGS)/whole exome sequencing (WES) data in FASTQ format. Preferably, in ProGeo-neo v2.0, several new features are provided. In addition to the identification of MHC-I neoantigens, the new version supports the prediction of MHC class II-restricted neoantigens, i.e., peptides up to 30-mer in length. Moreover, the source of neoantigens has been expanded, allowing more candidate neoantigens to be identified, such as in-frame insertion-deletion (indels) mutations, frameshift mutations, and gene fusion analysis. In addition, we propose two more efficient screening approaches, including an in-group authentic neoantigen peptides database and two more stringent thresholds. The range of candidate peptides was effectively narrowed down to those that are more likely to elicit an immune response, providing a more meaningful reference for subsequent experimental validation. Compared to ProGeo-neo, the ProGeo-neo v2.0 performed well based on the same dataset, including updated functionality and improved accuracy.

## 1. Introduction

Neoantigens are tumor-specific antigens (TSAs), which are expressed only in tumor cells and not in normal cells [1]. Neoantigens are often derived from a range of non-synonymous mutations, including single-nucleotide variation (SNV), insertion-deletion (INDEL), frameshift mutations, gene fusions, and structural variants [2]. Neoantigens are more immunogenic and more likely to elicit an immune response than common antigens because they do not undergo negative screening of the thymus. Mutated mRNA and protein sequences are generated after the transcribed and translation of non-synonymous genetic changes [3]. Mutated proteins are proteolytically cleaved into peptides, and then are naturally presented on the tumor cell surface by the MHC [4]. Eventually, the resultant complex can be recognized by cytotoxic T cells to generate an anti-tumor response on the tumor cell surface. The MHC genes, commonly known as human leukocyte antigen (HLA) in humans, are found on chromosome 6, including HLA-class I and HLA-class II. HLA-class I molecules are located on the surface of normal cells, which can be recognized by cytotoxic CD8^+^ T cell receptors. Most HLA-class II molecules are located on antigen-presenting cells (APCs), which can be recognized by cytotoxic CD4^+^ T cells [5]. The expression or non-expression of HLA-II molecules directly determines the development and intensity of the immune response. Changes in the expression levels of HLA class II molecules have also been associated with the development of certain autoimmune diseases, tumors, immunodeficiencies, and other disorders. Therefore, HLA-II molecules are of great value for immunological studies [6].

The discovery of neoantigens has made it possible to create personalized therapeutic cancer vaccines tailored for tumor patients. New evidence suggests that neoantigens can be recognized by the immune system and can be targeted to increase anti-tumor immunity [7]. Sahin’s team revealed in 2017 that cancer vaccines created by screening “neoantigens” from melanoma could activate CD8+ and CD4+ T cell proliferation, leading to tumor regression [8]. Furthermore, vaccines originally designed to target CD8+ T cells mediated a higher number of CD4+ T cells, raising concerns about the important role of HLA-II molecules in antitumor activity.

Personalized neoantigen vaccines are currently considered to be highly effective and safe in immunotherapy strategy. Therefore, timely and effective identification of neoantigens is in urgent need. Recently, with the development of next-generation sequencing (NGS) and bioinformatics techniques, various neoantigen prediction tools have been developed internationally. For example, specific tumor mutations were identified by integrating tumor mutation and expression data (DNA-seq and RNA-seq) in pVAC-seq [9]. As a machine learning approach, Neopepsee [10] was used to predict neoantigens by selecting nine strongly correlated features from fourteen neoantigen-related features. A neoantigen predictive pipeline, namely TSNAD, was developed by Zhejiang University in China, which can predict extracellular mutations in membrane proteins or mutant peptides presented by class I major histocompatibility complex molecules [11]. However, most neoantigen prediction pipelines published internationally are based on genomics. As far as we know, few pipelines can comprehensively predict neoantigens from various mutations, especially HLA class II-restricted neoantigens.

Advances in high-throughput NGS and mass spectrometry-based proteomics have led to the development of “proteogenomics”, which combines genomics and proteomics [12]. We previously reported on the construction of a proteogenomic predictive neoantigen (ProGeo-neo v1.0) pipeline, a tool that has been adopted by many groups [13]. In this paper, we present ProGeo-neo v2.0 with new improvements and features. The new version (i) updates all embedded tools to the latest version or replaces them with better-performing tools, (ii) adds the prediction of neoantigens from INDELs and gene fusions, (iii) increases the prediction of HLA class II-restricted neoantigens, (iv) starts the analysis from the WGS/WES data in FASTQ format, (v) information on mutations at the DNA level through intermediate files, (vi) is packaged as a one-stop software for ease to use. The source code of ProGeo-neo v2.0 is freely available at https://github.com/kbvstmd/ProGeo-neo2.0 (1 April 2022).

## 2. Material and Methods

### 2.1. Data

The same data used to build ProGeo-neo v1.0 were used to build ProGeo-neo v2.0. Jurkat’s WGS data is available at the NCBI’s Sequence Read Archive (SRA) study SRP101994 [14] (https://www.ncbi.nlm.nih.gov/sra/SRP101994 (16 March 2017)). Paired-end 200 bp sequencing RNA-seq data of Jurkat cell lines generated by Illumina HiSeq 2000, which was downloaded from the NCBI’s Gene Expression Omnibus (GEO) [15] repository under the accession number GSE45428 [16]. LC-MS/MS of Jurkat proteomics data may be downloaded via FTP from the PeptideAtlas data repository by accessing the following link: (http://www.peptideatlas.org/PASS/PASS00215 (15 April 2013)).

We downloaded human normal protein sequences in FASTA format from the Uniprot Database [17] (http://www.uniprot.org/ (14 May 2021)). The commonly contaminated laboratory protein sequences in FASTA format were downloaded from the common Repository of Adventitious Proteins (cRAP) (http://www.thegpm.org/crap/ (5 July 2021)).

### 2.2. Description of the ProGeo-Neo v2.0

**Figure 1:**
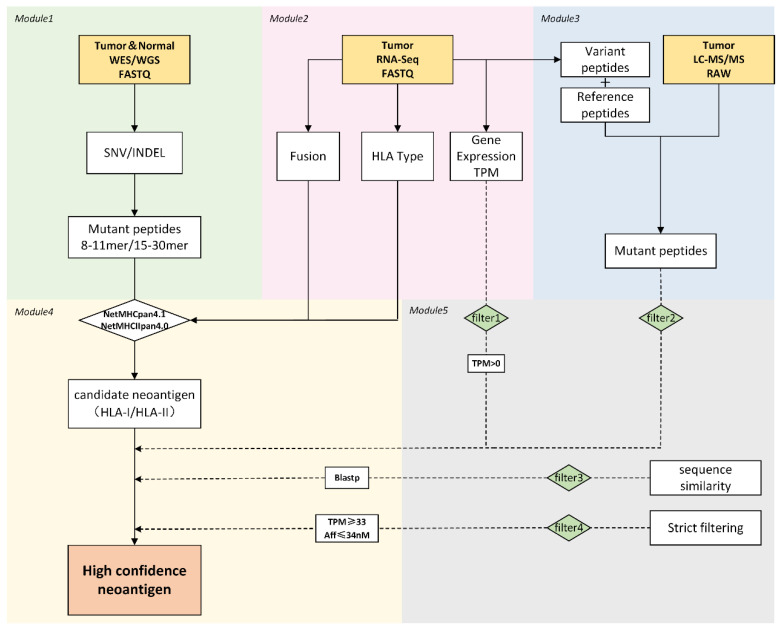
Workflow of ProGeo-neo v2.0. Including detection of SNV/INDEL based on tumor/normal WGS/WES data; HLA allele prediction, gene fusion detection, and gene expression detection based on tumor RNA-seq data; neoantigen screening by raw proteomics data (LC-MS/MS); neoantigen prediction (peptides-HLA class I/II); screening and filtering of candidate neoantigens.

As illustrated in Figure 1, the ProGeo-neo v2.0 workflow consists of five modules.

### 2.3. Module 1: Identification of SNV/INDEL Based on WGS/WES Data

Firstly, Trimmomatic (v0.38) [18] is used to trim and crop raw WGS reads and check quality results with FastQC (v0.11.5) [19] in ProGeo-neo v2.0. Then, BWA (v0.7.17) [20] is used for mapping all clean reads to a human reference genome (release hg38). SAMtools (v1.10) [21] is used to convert sequencing data formats from sequence alignment/map (SAM) to binary alignment/map (BAM) to save storage space and to sort the resulting bam files. GATK (v4.2.0.0) [22] is used to remove repetitive sequences and recalibrate the mass fraction of original bases. The Mutect2 module of GATK is used to call SNVs/INDELs. GATK’s VQSR and FilterMutectCalls modules are used for variant quality control.

### 2.4. Module 2: RNA-Seq Data Processing

The RNA-seq data were processed similarly to the WGS data to obtain the amino acid mutation. Amino acid changes caused by gene fusions are predicted by STAR-Fusion (v1.9.0) [23]. HLA-I alleles are inferred from RNA-seq data using OptiType (v1.3.5) [24] with default settings, and HLA-II alleles are inferred using HLAminer (v1.4) [25]. Transcripts expression is quantified by TPM level from RNA-seq data using Kallisto (v0.46.2) [26].

### 2.5. Module 3: Building Protein Database and MS Searching

RNA-seq data can provide a better reference proteomics dataset than WGS/WES, which typically has a lower read coverage. Therefore, we identify mutations at the protein level based on RNA-seq data. A customized searchable peptide database was built using human normal protein sequences, commonly contaminated laboratory protein sequences, and mutant protein sequences based on tumor RNA-seq data. MaxQuant (v2.0.1.0) [27] is then used to search raw proteomic spectra in a customized database to identify mutant peptides for protein-level validation of candidate neoantigens. The parameters of MaxQuant are set as follows: default parameters are used for peak generation; variable modifications including protein N-terminal acetylation, methionine oxidation, and Strict trypsin specificity were required to allow for up to two deletion cuts; the fixation modification is cysteine aminomethylation. The software automatically constructs the reverse sequence database as a decoy database. False discovery rate (FDR) thresholds for PSM, protein, peptides, and site were specified at 1%. The minimum required peptide length was set to 7.

### 2.6. Module 4: Neoantigen Prediction

**Figure 2:**
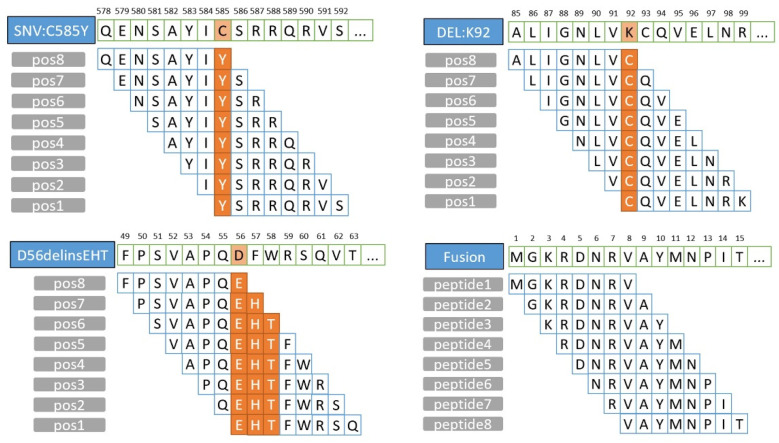
Schematic diagram of mutant peptides synthesis.

ANNOVAR (Date: 08 June 2020) [28] can be used for annotation to obtain mutation information at amino acid levels. Python (v3.7) is used to convert the amino acid mutation data into FASTQ sequences with lengths of 8–11-mer and 15–30-mer. This is completed as follows. First, the obtained amino acid mutation site information is used to match to human normal protein sequences by gene name, resulting in protein sequences containing amino acid mutation sites. Second, intercept a sequence of length k from the position k-1 in front of the mutation site with a stride of 1, ensuring that the site is included in each sequence. Then, apply this operation to all lengths. Finally, the mutant peptides are saved in FASTQ format for subsequent filtration. The mutant peptides interception operation is shown in Figure 2. The binding affinity of 8–11-mer peptides for HLA class I is predicted using the NetMHCpan (v4.1) [29] binding algorithm, and 15–30-mer peptides for HLA class II are predicted using the NetMHCIIpan (v4.0) [30]. The predicted HLA alleles and the mutated expressed peptides are used as input for the NetMHCpan 4.1/NetMHCIIpan 4.0 to estimate their binding affinities and predict neoantigens. FASTA files of amino acid mutant sequences resulting from gene fusions are also input to NetMHCpan for prediction. There are two methods to determine peptides-HLA binding affinity: half-maximum inhibitory concentration (IC50) and percentile rank scores (%Rank). The %Rank contains information relating to peptide-HLA binding events and previous steps in the biological antigen presentation pathway [31]. Therefore, candidate binders are selected based on %Rank first. Strongly (HLA-I: %Rank ≤ 0.5; HLA-II: %Rank ≤ 2) and weakly (HLA-I: 0.5 < %Rank ≤ 2; HLA-II: 2 < %Rank ≤ 10) bound predicted neoantigens are included in the subsequent analysis. However, the predicted IC50 value is retained for subsequent candidate peptides filtration.

### 2.7. Module 5: Neoantigen Filtration

The neoantigen filtration module is the most critical part of the ProGeo-neo v2.0. Four screening strategies are used in ProGeo-neo v2.0: (a) Gene expression level filtration: the gene expression detected in Module 2 is a filter to remove the peptides generated by mutations in unexpressed genes (TPM < 0), which could improve the accuracy of the final prediction. (b) Protein level filtration: the mutant peptides obtained after the MaxQuant library search in Module 3 were used as filters for candidate neoantigens. (c) Neoantigen database filtration: Sequence similarity filtering of data from in-house neoantigen database dbPepNeo v2.0 using the BLASTp tool [32,33]. A total of 801 high-confidence peptides (directly verified by experiment to elicit the immune response) and 251 medium-confidence peptides (verified by MS and WGS/WES) were used as filter libraries. (d) Strict threshold filtration for HLA class I restricted neoantigens: A more stringent threshold for HLA class I restricted neoantigens, (i.e., binding intensity ≤ 34 nM and tumor abundance ≥ 33 TPM) was proposed by Wells DK et al. based on their study was used to screen candidate peptides [34].

## 3. Results

### 3.1. Features Updated from ProGeo-Neo v1.0

In the first version of ProGeo-neo, candidate neoantigens are first identified from tumor genomic variants by using NetMHCpan-4.0, and then a customized database is created by analyzing RNA-seq data for variant peptides, and HLA alleles are inferred from RNA-seq data. In the whole process, several tools are applied, including Sickle, BWA, SAMtools, Bcftools, ANNOVAR, and OptiType. Some tools have been updated or deprecated with time, so in the new version, we update or replace them with other tools. The specific adjustment is as follows: (i) BWA, SAMtools, GATK, Kallisto, ANNOVAR, MaxQuant, and NetMHCpan are updated to a newer version. (ii) The Sickle tool has been replaced with Trimmomatic to remove low-quality reads, and the Bcftools tool has been replaced with the Mutect2 module of GATK to the call mutation. GATK’s Mutect2 has a complete analysis flow of calling somatic SNVs and Indels, which is more appropriate for ProGeo-neo v2.0 to run from tumor-normal paired WGS/WES sequencing data. (iii) New function was provided based on WGS/WES raw sequencing data analysis by combining GATK’s Mutect2 pipeline to call SNVs/INDELs. (iv) Added functionality for fusion genes being detected from RNA-seq data by STAR-Fusion. (v) Added functionality for predicting neoantigens based on peptide-HLA class II binding affinity by NetMHCIIpan.

There are two main improvements in the filtering of candidate peptides, which are as follows: we first used our group’s own authentic neoantigen peptides database for filtering, which effectively narrowed down the range of candidate peptides. Candidate peptides were obtained, which were more likely to elicit immune responses, providing a more meaningful reference for subsequent experimental validation. In addition, we have also adjusted the tumor abundance and peptides-HLA class I binding affinity cutoffs more strictly by referring to the suggested thresholds given in a recent study on neoantigen prediction [34]. These two strict thresholds have also been shown to indeed improve the quality of candidate neoantigens.

The information in our output results file is more detailed to give the user more references. Detected tumor abundance and peptides-HLA class I binding affinity have been added to the results file. Intermediate files with mutation information at the base level for mutant peptides are provided in ProGeo-neo v2.0. Further, result files are stored according to different mutation types, namely SNV, in-frame INS, in-frame DEL, frameshift, and fusion.

The source code of ProGeo-neo v2.0 is freely available at https://github.com/kbvstmd/ProGeo-neo2.0 (1 April 2022).

### 3.2. The Performance of ProGeo-Neo v2.0 on Jurkat Cell Line Data

In ProGeo-neo v2.0, the types of variants that support neoantigen prediction had been extended to include intra-frame indexing, frame-shift variants, and fusion. A total of 14,555 non-synonymous variants, 237 in-frame ins variants, 405 in-frame del variants, and 1123 frameshift variants were detected from the Jurkat WGS data. Fusion assay using STAR-Fusion detected a total of 62 amino acid mutation sequences arising from fusion genes. ProGeo-neo v2.0 predicted five HLA class I alleles from RNA-seq data from Jurkat, including HLA-A * 03:01, HLA-B * 07:02, HLA-B * 35:03, HLA-C * 07:02, HLA-C * 04:01; HLA class II alleles, two types: DRB4_ 0103, DRB4_0101.

**Figure 3:**
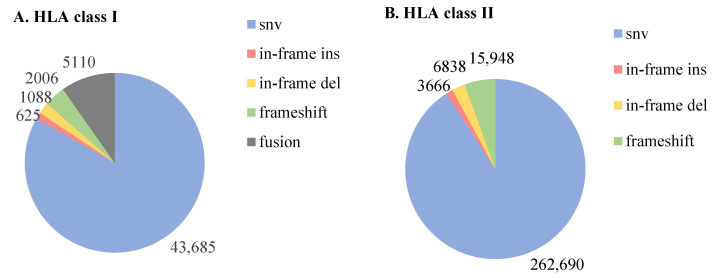
Source distribution of candidate neoantigens: (**A**). HLA class I binding neoantigens (8–11-mer) (**B**). HLA class II binding neoantigens (15–30-mer).

NetMHCpan predicted 376,671 mutant peptides of 8–11-mer length from 16,382 mutant sites based on Jurkat’s whole genome sequencing data. After NetMHCpan prediction, a total of 52,514 candidate neoantigens binding to HLA class I were obtained. These include 43,685 candidate neoantigens from missense mutations, 625 candidate neoantigens from in-frame ins mutations, 1088 candidate neoantigens from in-frame del mutations, 2006 candidate neoantigens from frameshift mutations, and 5110 candidate neoantigens from fusion mutations. (Figure 3A; Additional file 1: Table S1). Candidate neoantigens included 13,621 high-affinity peptides (%Rank ≤ 0.5) and 38,893 low-affinity peptides (0.5 < %Rank ≤ 2). NetMHCIIpan predicts 3,479,831 mutant peptides of 15–30-mer length from the same 16,382 mutant sites based on Jurkat’s WGS data. After NetMHCIIpan prediction, a total of 289,142 candidate neoantigens binding to HLA class II were obtained. These include 262,690 candidate neoantigens from missense mutations, 3666 candidate neoantigens from in-frame ins mutations, 6838 candidate neoantigens from in-frame del mutations, and 15,948 candidate neoantigens from frameshift mutations (Figure 3B; Additional file 2: Table S2). Candidate neoantigens included 47,144 high-affinity peptides (%Rank ≤ 2) and 241,998 low-affinity peptides (2 < %Rank ≤ 10).

**Figure 4:**
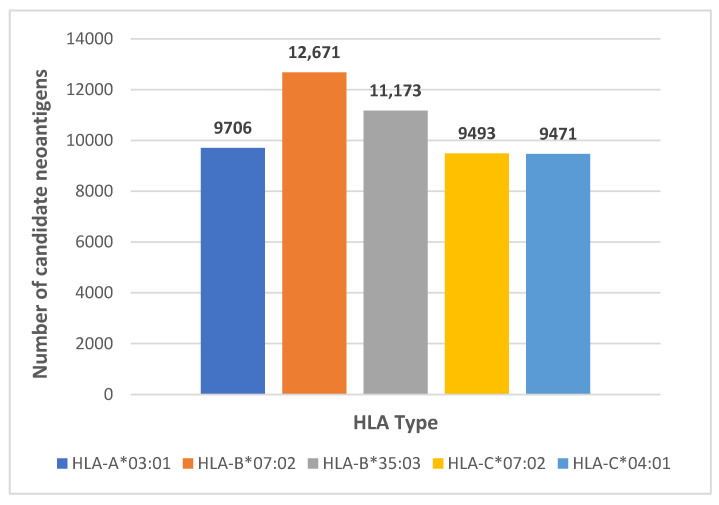
The number of predicted neoantigens bound to each HLA allele.

The number of neoantigens bound to each HLA class I allele was also counted, as shown in Figure 4. The number of the candidate neoantigens bound varies between alleles, ranging from 9471 to 12,671. We have also observed that some candidate neoantigens bind to different alleles simultaneously, and this type of candidate neoantigen is more valuable to study as it may apply to a broader range of individuals.

### 3.3. Performance Enhancements from ProGeo-Neo v1.0

The same data used to build ProGeo-neo v1.0 was used to build ProGeo-neo v2.0. In ProGeo-neo v2.0, 52,514 unfiltered HLA class I restricted neoantigen candidates were reported. In the original version of ProGeo-neo 36,835 neoantigens were reported. This demonstrated that, by extending support for additional variant types as well as prediction algorithms, we produced 42.6% more raw candidate neoantigens. Additionally, 289,138 HLA class II-restricted candidate neoantigens have been added.

Since ProGeo-neo v1.0 only supports the prediction of neoantigens bound to HLA class I molecules and mutation types are limited to non-synonymous mutations, we compare this part of ProGeo-neo v2.0’s results with the previous results. Firstly, the 636 HLA class I molecular binding candidate peptides obtained from protein level screening were compared for sequence similarity with the neoantigen peptides database established by our group using BLASTp. A total of 122 mutant peptides were found to resemble the authentic neoantigen sequences, with a matching ratio of 19.18%. (Additional file 3: Table S3). The matching ratio is defined as follows:

$$\mathrm{matching}\;\mathrm{ratio}\;=\frac{\mathrm{number}\;\mathrm{of}\;\mathrm{neoantigens}\;\mathrm{matched}\;\mathrm{to}\;\mathrm{the}\;\mathrm{database}}{\mathrm{number}\;\mathrm{of}\;\mathrm{neoantigens}\;\mathrm{being}\;\mathrm{compared}}$$

, with similarity threshold of 20 to 100

We have observed that most neoantigens have sequence similarity scores greater than 60, with the lowest score being 40. In general, the higher the sequence similarity, the greater the likelihood that the neoantigen will be recognized by the T-cell receptor. These 122 mutant peptides may be recognized by the TCR, and their immunogenicity can be further analyzed by experimental or clinical trials in leukemia patients. Second, ProGeo-neo v2.0 filtered candidate peptides by reference to the more stringent peptide thresholds for peptide-MHC class I, (i.e., binding intensity ≤ 34 nM and tumor abundance ≥ 33 TPM) proposed by Wells DK et al. A further 19 candidate peptides were left after the screening. We compared the 19 candidate peptides to the neoantigen database using BLASTp. Four peptides were found to resemble the neoantigen sequences in the database with a high degree of similarity, averaging 71.13 and a matching ratio of 21.05%. (Additional file 4: Table S4) Finally, to test the method’s accuracy in recognizing mutant peptides as neoantigens, we performed sequence similarity analysis on peptides obtained by NetMHCpan prediction but not filtered. It was found that 7514 peptides out of 52,514 candidate neoantigens had sequence similarity to peptides in the neoantigen database, with a matching ratio of 14.31%, significantly lower than our previous comparison probability.

We did sequence similarity analysis of the 655 peptides obtained after filtering at the ProGeo-neo v1.0 protein level with the neoantigen peptides database in the same way. Only 75 peptides were successfully matched, a matching ratio of 11.45%, significantly lower than our current result, even with the worst rate of 14.31%. Additionally, ProGeo-neo v2.0 had the best matching ratio of 21.05%. This shows a significant improvement in our accuracy rate compared to ProGeo-neo v1.0 and provides more robust support for subsequent validation. See Table 1 for details.

### 3.4. Screening Validity of ProGeo-Neo v2.0

**Figure 5:**
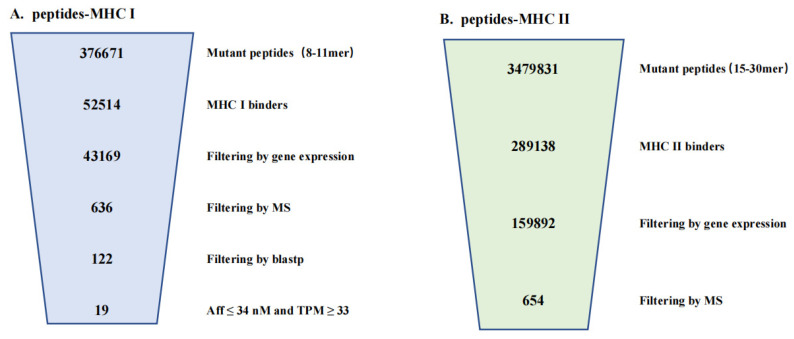
Results overview for the neoantigen discovery: (**A**). MHC class I binding neoantigens (8–11-mer) (**B**). MHC class II binding neoantigens (15–30-mer).

Cancer neoantigens are important targets for endogenous anti-tumor immune responses and cancer immunotherapies [35]. The accurate identification of cancer neoantigens is still a great challenge for neoantigen-related immunotherapy. Only a small percentage of candidate neoantigens predicted by existing bioinformatics tools elicit an immune response, suggesting that more research is needed to improve pipelines and algorithms for neoantigen prediction. The upgraded ProGeo-neo v2.0 combined with proteomics data in this study is more rigorous and credible than tools that use only genomic and transcriptomic data for prediction. ProGeo-neo v2.0 efficiently identifies and screens neoantigens and retains the more promising high-confidence peptides through four filters. The number of peptides obtained from the final identification was only a hundred, or even dozens (Figure 5A,B). Additionally, our results were proven to be reliable when compared with the validated neoantigen database, the highest matching ratio reached 21.05%. Such low-volume and reliable results are more beneficial to researchers, leading significantly to the reduction of time and cost at subsequent experimental validation.

Unfortunately, strict threshold versus true neoantigen peptide database filtering is only available for MHC class I molecularly restricted neoantigens. There are not many studies on MHC class II molecularly restricted neoantigens prediction, ProGeo-neo v2.0 provides a potentially useful first-line reference.

## 4. Discussion

ProGeo-neo v2.0 could provide one-stop neoantigen prediction based on original WGS/WES data of normal/tumor, tumor RNA-Seq data, and LC-MS/MS data. It updated or replaced most of the embedded tools with the latest version or a more appropriate tool in this release. It also supports the prediction of neoantigens bound to HLA class II molecules and provides analysis of in-frame indel, frameshift, and gene fusions. Compared with other neoantigen prediction pipelines, ProGeo-neo v2.0 has several advantages: (i) it can start the analysis from the raw FASTQ format data; (ii) it provides the analysis of SNVs, INDELs, and gene fusions; (iii) it can predict HLA-II restricted neoantigens, i.e., predicted neoantigen peptides lengths up to 30-mer; (iv) it provides a proteogenomic strategy that not only takes into account the neoantigens presented by HLA molecules, but also directly identifies these mutant peptides using MS data; (v) it uses a validated neoantigen peptides database for sequence similarity screening; (vi) more stringent tumor abundance and binding affinity thresholds referred to recent studies are used for neoantigen screening. Expanding the mutational origin of predicted neoantigens could allow more peptides with the potential to become neoantigens to enter subsequent studies. The software is constructed based on the genomic and proteomic data from the Jurkat leukemia cell line but applies to the prediction of neoantigens in other individual solid tumors.

Controlling false-positive mutations is a major challenge for neoantigen identification. In a recent study by the Tumor Neoantigen Selection Alliance (TESLA), the overall positivity rate of 25 teams predicting neoantigens on the same standard dataset was only 6.1%. Many factors affect the positive rate of neoantigen prediction, such as the limitation of the algorithm, the limitation of sequencing technology, and the heterogeneity of the tumor. ProGeo-neo v2.0 was updated to use GATK4 Mutect2 for calling SNVs and Indels to better filter out germline mutation sites and reduce the false-positive rate. In addition, the most important improvement is the presentation of two new methods for screening neoantigens in ProGeo-neo v2.0, retaining mass spectrometry-based proteogenomics in the meantime. The addition of two new neoantigen screening methods has been shown to improve the accuracy and usability of neoantigen prediction results significantly. Effective neoantigen prediction relies on understanding the parameters governing epitope immunogenicity. ProGeo-neo v2.0 performs neoantigen filtration by adjusting thresholds for immune-related parameters and identifies candidate neoantigens with higher quality than existing processes that predict neoantigens using genomic data alone. Additionally, the candidate new peptides obtained by screening using actual neoantigen databases are more credible.

However, the successful elicitation of an immune response to the neo-peptide is a complex process that requires further investigation of T cell receptor (TCR)-pMHC interactions, which could be considered for further screening. In addition, neoantigens from non-coding regions should also be of interest. We will consider these aspects in our future updates.

## 5. Conclusions

One-stop neoantigen prediction and screening based on original WGS/WES, RNA-Seq, and LC-MS/MS data were provided in ProGeo-neo v2.0. ProGeo-neo v2.0 is an integrated software written in the Python programming language (v3.8) and requires standard third-party software. The software contains five main modules, namely: identification of SNVs/INDELs based on WGS/WES data; RNA-seq data processing for detecting gene fusions, accessing to HLA typing and gene expression; building customized protein database and MS searching; prediction of neoantigens by NetMHCpan/NetMHCIIpan; filtering candidate neoantigens by four methods.

The software is divided into five toolkits based on five modules. Before running the toolkit, the user needs to configure the software paths and parameters. This step is of great importance. After setting up the configuration, the user can run the pipeline by executing the command line. More detailed information can be found in the user manual.

## Figures and Tables

**Table 1:** Performance comparison between ProGeo-neo v2.0 and ProGeo-neo v1.0.

	Mutant Peptides	MHC I Binders	Filtering by Gene Expression	Filtering by MS	Aff ≤ 34 nM TPM ≥ 33
ProGeo-neo v2.0	376,671	52,514	43,169	636	19
**Matching ratio**		14.31%		19.18%	21.05%
ProGeo-neo v1.0	373,046	36,835	30,142	655	
**Matching ratio**				11.45%	

## Data Availability

The data used in this study are described in detail in *2.1 Data*. The source code of ProGeo-neo v2.0 is freely available at https://github.com/kbvstmd/ProGeo-neo2.0 (1 April 2022).

## Supplementary Materials

The following supporting information can be downloaded at: https://www.mdpi.com/article/10.3390/genes13050783/s1, Table S1. Candidate HLA class I restricted neoantigens. Table S2. Candidate HLA class II restricted neoantigens. Table S3. Results of HLA class I restricted neoantigens BLASTp after protein level filtering. Table S4. Results of HLA class I restricted neoantigens BLASTp after two strict thresholds screening.

## References

[1] Blass E., Ott P.A. (2021). Advances in the development of personalized neoantigen-based therapeutic cancer vaccines. Nat. Rev. Clin. Oncol..

[2] Ebrahimi N., Akbari M., Ghanaatian M., Roozbahani Moghaddam P., Adelian S., Borjian Boroujeni M., Yazdani E., Ahmadi A., Hamblin M.R. (2021). Development of neoantigens: From identification in cancer cells to application in cancer vaccines. Expert Rev. Vaccines.

[3] Supabphol S., Li L., Goedegebuure S.P., Gillanders W.E. (2021). Neoantigen vaccine platforms in clinical development: Understanding the future of personalized immunotherapy. Expert Opin. Investig. Drugs.

[4] Efremova M., Finotello F., Rieder D., Trajanoski Z. (2017). Neoantigens Generated by Individual Mutations and Their Role in Cancer Immunity and Immunotherapy. Front. Immunol..

[5] Algarra I., Garrido F., Garcia-Lora A.M. (2021). MHC heterogeneity and response of metastases to immunotherapy. Cancer Metastasis Rev..

[6] Shao X.M., Bhattacharya R., Huang J., Sivakumar I., Karchin R. (2020). High-Throughput Prediction of MHC Class I and II Neoantigens with MHCnuggets. Cancer Immunol. Res..

[7] Yarchoan M., Johnson B.A., Lutz E.R., Laheru D.A., Jaffee E.M. (2017). Targeting neoantigens to augment antitumour immunity. Nat. Rev. Cancer.

[8] Sahin U., Derhovanessian E., Miller M., Kloke B.P., Simon P., Löwer M., Bukur V., Tadmor A.D., Luxemburger U., Schrörs B. (2017). Personalized RNA mutanome vaccines mobilize poly-specific therapeutic immunity against cancer. Nature.

[9] Hundal J., Carreno B.M., Petti A.A., Linette G.P., Griffith O.L., Mardis E.R., Griffith M. (2016). pVAC-Seq: A genome-guided in silico approach to identifying tumor neoantigens. Genome Med..

[10] Kim S., Kim H.S., Kim E., Lee M.G., Shin E.C., Paik S., Kim S. (2018). Neopepsee: Accurate genome-level prediction of neoantigens by harnessing sequence and amino acid immunogenicity information. Ann. Oncol. Off. J. Eur. Soc. Med. Oncol..

[11] Zhou Z., Lyu X., Wu J., Yang X., Wu S., Zhou J., Gu X., Su Z., Chen S. (2017). TSNAD: An integrated software for cancer somatic mutation and tumour-specific neoantigen detection. R. Soc. Open Sci..

[12] Verma A., Halder A., Marathe S., Purwar R., Srivastava S. (2020). A proteogenomic approach to target neoantigens in solid tumors. Expert Rev. Proteom..

[13] Li Y., Wang G., Tan X., Ouyang J., Zhang M., Song X., Liu Q., Leng Q., Chen L., Xie L. (2020). ProGeo-neo: A customized proteogenomic workflow for neoantigen prediction and selection. BMC Med. Genom..

[14] Gioia L., Siddique A., Head S.R., Salomon D.R., Su A.I. (2018). A genome-wide survey of mutations in the Jurkat cell line. BMC Genom..

[15] Barrett T., Troup D.B., Wilhite S.E., Ledoux P., Rudnev D., Evangelista C., Kim I.F., Soboleva A., Tomashevsky M., Marshall K.A. (2009). NCBI GEO: Archive for high-throughput functional genomic data. Nucleic Acids Res..

[16] Sheynkman G.M., Shortreed M.R., Frey B.L., Smith L.M. (2013). Discovery and mass spectrometric analysis of novel splice-junction peptides using RNA-Seq. Mol. Cell. Proteom. MCP.

[17] UniProt Consortium (2015). UniProt: A hub for protein information. Nucleic Acids Res..

[18] Bolger A.M., Lohse M., Usadel B. (2014). Trimmomatic: A flexible trimmer for Illumina sequence data. Bioinformatics.

[19] Andrews S. (2010). FastQC: A Quality Control Tool for High Throughput Sequence Data. https://www.bioinformatics.babraham.ac.uk/projects/fastqc/.

[20] Li H., Durbin R. (2010). Fast and accurate long-read alignment with Burrows-Wheeler transform. Bioinformatics.

[21] Li H. (2011). A statistical framework for SNP calling, mutation discovery, association mapping and population genetical parameter estimation from sequencing data. Bioinformatics.

[22] McKenna A., Hanna M., Banks E., Sivachenko A., Cibulskis K., Kernytsky A., Garimella K., Altshuler D., Gabriel S., Daly M. (2010). The Genome Analysis Toolkit: A MapReduce framework for analyzing next-generation DNA sequencing data. Genome Res..

[23] Haas B., Dobin A., Stransky N., Bo L., Xiao Y., Tickle T., Bankapur A., Ganote C., Doak T., Pochet N. (2017). STAR-Fusion: Fast and Accurate Fusion Transcript Detection from RNA-Seq. BioRxiv.

[24] Szolek A., Schubert B., Mohr C., Sturm M., Feldhahn M., Kohlbacher O. (2014). OptiType: Precision HLA typing from next-generation sequencing data. Bioinformatics.

[25] Warren R.L., Choe G., Freeman D.J., Castellarin M., Munro S., Moore R., Holt R.A. (2012). Derivation of HLA types from shotgun sequence datasets. Genome Med..

[26] Bray N.L., Pimentel H., Melsted P., Pachter L. (2016). Near-optimal probabilistic RNA-seq quantification. Nat. Biotechnol..

[27] Tyanova S., Temu T., Cox J. (2016). The MaxQuant computational platform for mass spectrometry-based shotgun proteomics. Nat. Protoc..

[28] Wang K., Li M., Hakonarson H. (2010). ANNOVAR: Functional annotation of genetic variants from high-throughput sequencing data. Nucleic Acids Res..

[29] Jurtz V., Paul S., Andreatta M., Marcatili P., Peters B., Nielsen M. (2017). NetMHCpan-4.0: Improved Peptide-MHC Class I Interaction Predictions Integrating Eluted Ligand and Peptide Binding Affinity Data. J. Immunol..

[30] Karosiene E., Rasmussen M., Blicher T., Lund O., Buus S., Nielsen M. (2013). NetMHCIIpan-3.0, a common pan-specific MHC class II prediction method including all three human MHC class II isotypes, HLA-DR, HLA-DP and HLA-DQ. Immunogenetics.

[31] Reynisson B., Alvarez B., Paul S., Peters B., Nielsen M. (2020). NetMHCpan-4.1 and NetMHCIIpan-4.0: Improved predictions of MHC antigen presentation by concurrent motif deconvolution and integration of MS MHC eluted ligand data. Nucleic Acids Res..

[32] Tan X., Li D., Huang P., Jian X., Wan H., Wang G., Li Y., Ouyang J., Lin Y., Xie L. (2020). dbPepNeo: A manually curated database for human tumor neoantigen peptides. Database J. Biol. Databases Curation.

[33] McGinnis S., Madden T.L. (2004). BLAST: At the core of a powerful and diverse set of sequence analysis tools. Nucleic Acids Res..

[34] Wells D.K., van Buuren M.M., Dang K.K., Hubbard-Lucey V.M., Sheehan K.C.F., Campbell K.M., Lamb A., Ward J.P., Sidney J., Blazquez A.B. (2020). Key Parameters of Tumor Epitope Immunogenicity Revealed Through a Consortium Approach Improve Neoantigen Prediction. Cell.

[35] Chen I., Chen M.Y., Goedegebuure S.P., Gillanders W.E. (2021). Challenges targeting cancer neoantigens in 2021: A systematic literature review. Expert Rev. Vaccines.

